# Breaking the Efflux Barrier: P-Glycoprotein and Emerging Strategies to Overcome Multidrug Resistance in Cancer

**DOI:** 10.3390/cancers18132047

**Published:** 2026-06-24

**Authors:** Alina Crenguța Nicolae, Carmen Adella Sîrbu, Ion-Bogdan Dumitrescu, Elena Moroşan, Cristina Manuela Drăgoi

**Affiliations:** 1 Department of Biochemistry, Faculty of Pharmacy, Carol Davila University of Medicine and Pharmacy, 020956 Bucharest, Romania; alina.nicolae@umfcd.ro (A.C.N.); cristina.dragoi@umfcd.ro (C.M.D.); 2 Clinical Neuroscience Department, Carol Davila University of Medicine and Pharmacy, 050474 Bucharest, Romania; 3 Academy of Romanian Scientists, 050045 Bucharest, Romania; 4 Department of Physics and Informatics, Faculty of Pharmacy, Carol Davila University of Medicine and Pharmacy, 020956 Bucharest, Romania; 5 Department of Clinical Laboratory and Food Safety, Faculty of Pharmacy, Carol Davila University of Medicine and Pharmacy, 020956 Bucharest, Romania; elena.morosan@umfcd.ro

**Keywords:** multidrug resistance (MDR), P-glycoprotein (P-gp; ABCB1), ABC transporters, drug efflux, cancer therapy, nanotechnology-based drug delivery, natural compounds, oxidative stress, tumor microenvironment, pharmacological modulation

## Abstract

Multidrug resistance (MDR) remains a major obstacle in cancer therapy, driving treatment failure and disease progression across diverse malignancies. A key determinant of MDR is the overexpression of ATP-binding cassette (ABC) transporters, particularly P-glycoprotein (P-gp/ABCB1), which actively effluxes structurally diverse chemotherapeutic agents and reduces their intracellular accumulation. Despite extensive investigation, clinically effective strategies to overcome P-gp-mediated resistance remain limited. This review provides a comprehensive analysis of the molecular mechanisms underlying P-gp function, including its structural organization, regulation of expression, and role in cellular drug disposition. We highlight the interplay between P-gp activity, oxidative stress, metabolic reprogramming and the tumor microenvironment, emphasizing the complexity of MDR as a dynamic and adaptive process. Emerging therapeutic approaches targeting P-gp-mediated resistance are also discussed, including natural bioactive compounds, nanotechnology-based drug delivery systems, polymeric carriers and novel anticancer agents designed to evade efflux mechanisms. Integrating mechanistic insights with advanced pharmacological strategies may improve intracellular drug retention and therapeutic efficacy. A deeper understanding of P-gp-driven MDR is essential for the development of effective interventions aimed at overcoming drug resistance and improving clinical outcomes in cancer patients.

## Simple Summary

Cancer treatment is often limited by the ability of tumor cells to develop resistances to multiple drugs. One of the main mechanisms behind this phenomenon is the overexpression of P-glycoprotein, a membrane protein that acts as a pump and removes anticancer drugs from the cell before they could exert their effect. As a result, treatments become less effective and the disease may progress. This review explains how P-glycoprotein contributes to multidrug resistance and highlights the complex biological processes involved, including changes in cellular metabolism and oxidative stress. It also presents several emerging strategies designed to overcome this resistance, such as natural compounds, polymer-based systems, nanotechnology-driven drug delivery and more recent anticancer agents that are less affected by drug efflux. A better understanding of these mechanisms may help improve current therapies and support the development of more effective treatments for patients with drug-resistant cancers.

## 1. Introduction

Cancer is a biologically heterogeneous and evolutionarily dynamic disease characterized by the progressive accumulation of genetic, epigenetic and metabolic alterations that disrupt cellular homeostasis. Malignant transformation results from the deregulation of tightly controlled processes governing proliferation, apoptosis, differentiation and genomic stability. These alterations enable tumor cells to acquire hallmark capabilities, including sustained proliferative signaling, resistance to cell death, replicative immortality, metabolic reprogramming as well as an invasive potential [1,2,3].

From a clinical standpoint, neoplasms are broadly classified as benign or malignant based on their biological behavior and molecular characteristics. Benign tumors are typically well-circumscribed, slow-growing lesions devoid of invasive or metastatic capacity. Malignant neoplasms, by contrast, are defined by genomic instability, elevated mitotic activity, local tissue invasion, and the propensity to disseminate to distant organs via hematogenous or lymphatic routes, giving rise to metastatic lesions in sites such as the liver, brain, and bone. The progression from a localized neoplasm to a fully invasive and metastatic malignancy is orchestrated by the sequential accumulation of molecular alterations, including oncogene activation (most notably MYC), loss of tumor suppressor function such as that mediated by TP53, aberrant activation of key signaling cascades, and dynamic adaptation to the surrounding tumor microenvironment [4,5,6].

Despite remarkable advances in chemotherapy, targeted therapies, immunotherapy, and precision oncology, cancer continues to represent a leading cause of morbidity and mortality on a global scale. A fundamental obstacle to the long-term success of systemic anticancer treatment is the emergence of multidrug resistance (MDR), a complex and multifactorial phenomenon that critically undermines therapeutic efficacy and durable disease control. MDR may be intrinsic, present prior to any therapeutic exposure, or acquired as a consequence of selective pressure exerted by cytotoxic agents. In both contexts, resistant cellular subpopulations evade elimination, undergo clonal expansion, and ultimately drive tumor relapse and disease progression, representing one of the most pressing unresolved challenges in clinical oncology [7,8].

In this context, chronobiology has emerged as a scientifically grounded and clinically relevant perspective for refining cancer prognosis and optimizing therapeutic strategies. The circadian clock, an evolutionarily conserved molecular oscillator governed by a transcription–translation feedback loop operating on an approximately 24 h cycle, coordinates a broad spectrum of physiological processes, including cell cycle progression, DNA damage repair, apoptosis, and immune surveillance, that are directly implicated in tumor biology [9,10,11,12]. Disruption of circadian rhythmicity at both the systemic and intracellular levels has been associated with accelerated tumor growth, enhanced metastatic dissemination, and diminished sensitivity to conventional cytotoxic agents. Conversely, the incorporation of chronobiological parameters into oncological decision-making, encompassing the optimization of drug administration timing, circadian biomarker profiling, and patient stratification according to individual chronotype, holds considerable potential for improving prognostic precision and therapeutic outcomes, particularly in the context of MDR modulation [13,14,15].

Complementary to chronobiological approaches, nutritional modulation has gained increasing scientific recognition as a meaningful adjunctive strategy in cancer prognosis and management [16,17,18]. A growing body of evidence demonstrates that dietary patterns, specific macro- and micronutrient profiles, and bioactive phytochemical compounds exert substantial influence over the molecular networks governing tumor initiation, clonal evolution, and resistance to therapy. Nutritional status modulates the composition and functional state of the tumor microenvironment, regulates inflammatory and oxidative stress signaling, reshapes epigenetic landscapes, and can directly affect the expression and activity of efflux transporters central to MDR [19,20,21,22]. In this regard, targeted nutritional interventions, encompassing caloric restriction mimetics, ketogenic dietary protocols, and the supplementation of plant-derived compounds with documented anti-tumorigenic and transporter-modulatory properties, constitute a mechanistically plausible and clinically accessible avenue for influencing cancer prognosis and potentially augmenting the efficacy of established anticancer regimens [23,24,25].

Historically, drug resistance was primarily attributed to mutations affecting drug targets, enhanced DNA repair capacity or defective apoptotic signaling [26,27,28]. However, contemporary evidence indicates that MDR represents a multifactorial adaptive response involving coordinated genomic, transcriptional, metabolic and membrane transport alterations. Among these mechanisms, the overexpression of ATP-binding cassette (ABC) transporters, particularly P-glycoprotein (P-gp, encoded by ABCB1), plays a central role. P-gp functions as an ATP-dependent efflux pump that actively exports structurally diverse chemotherapeutic agents, thereby reducing intracellular drug accumulation below cytotoxic thresholds [29,30].

Importantly, multidrug resistance is increasingly recognized as a dynamic, metabolically integrated and adaptive systems-level response to therapeutic stress, rather than a purely static genetic event. Tumor cells exposed to chemotherapeutic agents undergo profound metabolic and transcriptional reprogramming, including alterations in redox homeostasis, mitochondrial function, ATP turnover and membrane lipid composition, which create a permissive environment for the up-regulation of membrane transport systems that actively limit intracellular drug accumulation [31,32,33,34,35].

In this context, experimental data derived from in vitro and in vivo models have demonstrated that pharmacological exposure may modulate both oxidative stress parameters and P-gp expression [36,37,38,39,40,41]. Furthermore, experimental animal models remain essential for understanding the biochemical and pharmacological mechanisms underlying metabolic disorders and their systemic consequences [42,43,44,45,46,47,48,49,50,51,52]. Variations in systemic and tissue-specific oxidative biomarkers have been correlated with transporter regulation, thus supporting the existence of a functional interplay between cellular redox state and ABC transporter dynamics [53,54,55,56,57].

Among these systems, ABC transporters represent a central determinant of chemotherapeutic failure. These transmembrane proteins utilize ATP hydrolysis to actively expel structurally diverse xenobiotics across the plasma membrane. Of particular relevance in oncology is P-gp (ABCB1), one of the most extensively studied efflux pumps associated with resistance to anthracyclines, taxanes, vinca alkaloids and multiple other targeted agents. P-gp overexpression reduces intracellular drug concentrations below cytotoxic thresholds, thereby enabling tumor cell survival despite adequate systemic drug exposure [7,58,59,60]. Notably, P-gp regulation appears to be tightly interconnected with cellular metabolic status and oxidative signaling. Experimental studies have demonstrated that pharmacological modulation of oxidative stress parameters may influence P-gp expression and activity, highlighting a functional crosstalk between redox homeostasis and ABC transporter dynamics. These observations further support the concept that MDR is sustained by coordinated biochemical adaptations rather than isolated molecular alterations [40,54,61,62,63].

Given the pivotal contribution of P-gp to therapeutic resistance, a comprehensive understanding of its structural organization, regulatory mechanisms, and functional dynamics is essential for the development of effective MDR-modulating strategies.

### Literature Search Strategy

This article is a narrative review. Relevant literature was identified through structured searches of PubMed/MEDLINE, Scopus and Web of Science, complemented by manual screening of the reference lists of key articles. Search terms combined the concepts ‘P-gp’, ‘ABCB1’, ‘MDR1’, ‘multidrug resistance’, ‘drug efflux’ and ‘ABC transporters’ with strategy-specific descriptors (‘inhibitor’, ‘natural compound’, ‘nanoparti-cle/nanomedicine’, ‘chronotherapy/circadian’, ‘epigenetic’, ‘tumour microenvironment’, ‘precision oncology’). We considered peer-reviewed articles published in English, prioritising the period 2000–2025 while retaining seminal earlier studies of mechanistic importance. Preference was given to original research, randomized controlled trials, and authoritative reviews; conference abstracts, while non-peer-reviewed sources were excluded. As the objective was an integrative, conceptual synthesis spanning heterogeneous study types rather than a quantitative meta-analysis, a narrative (rather than systematic/PRISMA) framework was adopted, with study selection guided by relevance and methodological quality. We acknowledge that this approach carries a potential for se-lection bias, which we have sought to mitigate by explicitly grading the level of evidence underlying each strategy discussed.

## 2. The Phenomenon of Multi-Drug Resistance in Cancer

### 2.1. ATP Binding Cassette (ABC) Transporters

Discovered 40 years ago, P-gp is now, more than ever, being studied to understand the possibility of decreasing resistance in cancer. Overexpression of P-gp has been detected in over 50% of NCI-60 tumor lines, including melanoma, CNS tumors and a good proportion of renal and colon carcinomas. Reduced response to chemotherapy or well-below average clinical outcomes were also observed in antineoplastic therapy of solid or hematological tumors, where overexpression of P-gp has been identified [64,65].

Currently, the FDA and EMA require mandatory presence of interactions with P-gp and other important membrane transporters in the clinical investigation file of a potential innovative drug prior to approval. This is an indication of the significance of this efflux pump in the development of novel therapeutic strategies. Therefore, understanding the structure, activity and interaction at the molecular level of these membrane transporters with xenobiotics is of extreme importance at present, aimed at the possible interaction prediction of a new drug with these pumps, focusing on the synthesis of new compounds with inhibitory potential over them that facilitate drug action at the cellular level and decrease the MDR phenomenon or on the development of new therapeutic strategies to avoid efflux of therapeutic agents [66,67,68,69,70,71,72].

ABCs are a superfamily of proteins present at all taxonomic levels from prokaryotes to humans. P-gp is part of the ABC transporter family and is the cellular product of the MDR1 gene. It is also known as ABCB1, MDR1, PGY1, and CD243 (cluster of differentiation 243). Physiologically, it is expressed in tissues that influence the uptake, distribution and excretion of xenobiotics, but also in other tissues such as the adrenal glands, placenta or cancer cells. P-gp was originally thought to have implications in modulating cell permeability, hence the acronym P [29,60,73]. The involvement of P-gp in the MDR phenomenon in cancer was discovered by Juliano and Ling in drug-resistant malignancies [74]. They showed that despite the fact that some cancer cells were selected for resistance to a singular cytotoxic agent, they developed cross-resistance over time to a broad spectrum of antineoplastic compounds with different structures. Based on its ability to confer drug resistance to cancer cells, it has been successfully cloned and used in the laboratory to study its structure, mechanism of action and various interaction potentials [74]. At plasma membrane level, P-gp localizes within cholesterol-rich microdomains. These areas of the membrane are characterized by lower fluidity compared to the rest of the membrane, the reason being the increased cholesterol content and the interactions of cholesterol with saturated lipids [74].

Although P-gp is mainly localized to the cell membrane surface for its efflux function, it also has intracellular sites. Using immunofluorescence methods, it has been possible to prove the existence of this transporter protein also at the level of numerous cellular organelles, such as the endoplasmic reticulum, Golgi apparatus, lysosomes, endocytosomes and proteasome [75,76,77,78]. The varied localizations at cellular level provides information on the sites of synthesis (endoplasmic reticulum), modification (Golgi), transport (Golgi and endozymes) and degradation (lysosomes and proteasome) of P-gp. These organelles take turns in participating in the P-gp life cycle through the functions mentioned above [79,80,81].

**Figure 1:**
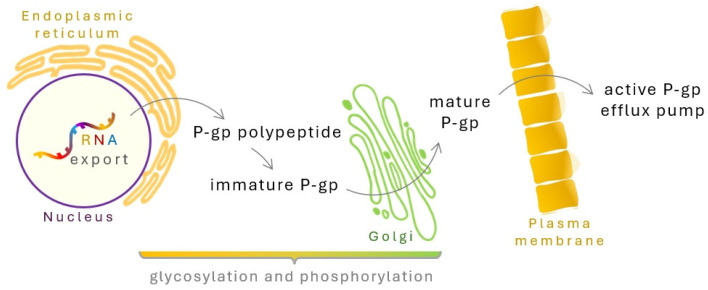
Biosynthesis, intracellular transport and membrane recycling of P-gp [82,83,84].

Given the relatively high half-life of P-gp (14–17 h), endoplasmic reticulum synthesis activity is not very intense. Transport time and plasma membrane binding of P-gp may be accelerated in P-gp-mediated MDR, when overexpression is influenced by the acceleration of all processes, including synthesis. The synthesis, transport and recycling processes are depicted in Figure 1 [82,83,84].

### 2.2. P-gp Structure

P-gp has a molecular weight of 170 KDa and consists of 1280 amino acids. It includes an N-terminal glycosylation core of 10–15 KDa, which is found in the first extracellular loop. The 1280 amino acids are arranged in two symmetrical regions linked by a bridge of about 60 amino acids, which contains phosphorylated serine residues. Each region contains a hydrophobic domain, with 6 transmembrane segments, followed by a hydrophilic domain that forms an ATP binding site, called NBDs (nucleotide binding domains). The C-terminal part is 65% similar to the N-terminal segment. There are 2 ATP binding sites, NBD1 and NBD2. They are not functionally identical but are essential for providing the transport function of P-gp. The difference is that the NBD1 site has both ATP-ase activity and GTP-ase activity. ATP-ase binding activity is the functional evidence for specific xenobiotic binding, so it can be used in the study of P-gp interaction with different substrates. Both halves of the P-gp structure have a binding domain where the substrate can bind, which is located intramembranely. The phosphorylation domain of P-gp plays a minor but important role in the control of ATP-base activity in NBD1 [85].

In inward-facing, nucleotide-free conformation, the catalytic nucleotide-binding domains are widely separated (~40 Å); this geometry, however, represents one extreme of a dynamic conformational ensemble. Upon ATP binding, the NBDs converge into a catalytically competent sandwich dimer, reorienting the transmembrane drug-binding cavity toward the extracellular milieu and effecting substrate extrusion, consistent with the conformational cycle resolved by single-particle cryo-electron microscopy [86,87,88].

#### 2.2.1. P-gp Biosynthesis

P-gp is initially synthesized at endoplasmic reticulum level in an immature form, not yet able to perform its functions. Glycosylation and phosphorylation are the processes by which P-gp acquires its specific efflux pump functions through structural changes. Subsequently, it will become scaffolded at the plasma membrane surface, in the shelf regions, where it has favorable tropism for expression (Figure 1) [89,90].

#### 2.2.2. Glycosylation

The molecular weight of P-gp varies between 130 and 180 KDa, depending on the species and cell type. P-gp is synthesized as a glycosylated precursor with a molecular weight between 120 and 140 KDa. In human P-gp, all oligosaccharide chains are known to be N-glycosylated, but the sequence and nature of the carbohydrates are still unknown. As for the binding sites, their number and position differ from species to species, but they are always found in the first loop. It is worth noting that drug transport activity and ATP-ase activity are not glycosylation-dependent. A number of mutations with altered glycosylation sites prove that drug resistance is not directly influenced by the glycosylation process. It is suggested that glycosylation would be a factor influencing the proper folding and binding of P-gp to the plasma membrane surface, but would not protect the protein from proteolytic cleavage, as the 14–17 h half-life of P-gp is not affected [90].

#### 2.2.3. Phosphorylation

Phosphorylation of P-gp is essential for its maturation process. It is enhanced by protein kinases, whereas phosphatases increase dephosphorylation. Thus, P-gp phosphorylation is stimulated by protein kinase inducers and decreased by protein kinase inhibitors. P-gp phosphorylation is correlated with drug resistance, either by affecting stability or by altering the rate of transport. Although the phosphorylation process does not play a key role in drug resistance, it has been suggested that the binding affinity of P-gp to some substrates may depend on the degree of phosphorylation. Even though there are about 40 different binding sites at the level of the P-gp structure, there are 4 serine residues that can be phosphorylated by protein kinase A and C, which could be found clustered in the region called “Linker”, present in the central part of the glycoprotein. Thus, protein kinases act at the linker region [60,79].

### 2.3. Functions and Mechanisms of P-gp

Although P-gp is currently intensely discussed as an important mechanism for the development of MDR in cancer, it is a protein structure widely found in the human body that performs numerous functions necessary to maintain general homeostasis. It is noteworthy that in its propelling activity, most of the compounds it transports are hydrophobic in nature. P-gp has various roles in the absorption, distribution, metabolism and excretion of chemical agents. It regulates the bioavailability and distribution of some drugs. Overexpression of P-gp in the gut can result in decreased absorption of drugs that are P-gp substrates, with an associated decrease in bioavailability. In parallel, the under expression of P-gp in the gut can lead to excess accumulation of drugs in the blood, with an implicit increase in toxic potential. It also plays a role in detoxifying the body by excreting toxic or xenobiotic metabolites into the urine, bile or intestinal lumen. Nevertheless, it has a role in the transport of hormones, especially pancreatic, thyroid, adrenocortical, steroids and somatostatin. It plays an active role in the migration of dendritic cells and causes translocation of phospholipids, through an activity similar to a translocase on short lipid chains. In this way, it acts on phosphatidylcholine, facilitating its transfer from the inner to the outer side of the membrane, an ATP-dependent process. In addition, it is involved in cytotoxic processes and it is expressed on the surface of natural killer cells [80,91].

P-gp’s mechanism of action is based on its ATP-dependent efflux pump activity, as its overexpression leads to low therapeutic concentrations and rapid drug clearance. These effects are significantly reduced either by intracellular ATP depletion or by mutations in the two halves of the protein and, nevertheless, mutations in the transmembrane segments or cytosolic loops of P-gp that alter substrate specificity and binding of different drugs. Studies of mutations and chemical modifications of transmembrane segments show that they interact with the catalytic sites of P-gp. Although each half exhibits its own activity, their simultaneous expression and interactions between the two are essential for functioning. Mutations at NBD sites have resulted in complete inactivation of pump function, providing clear evidence for the role of these sites. There are 3 drug-dependent phases of ATPase activity associated with P-gp: basal phase (no drug present), drug-activated phase (during interaction with a moderate dose of drug), inhibited phase (when the drug dose becomes too high) [92,93,94].

**Figure 2:**
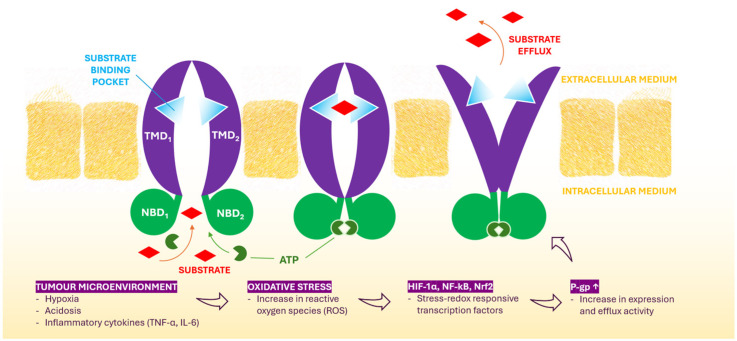
Mechanism and regulation of P-glycoprotein (P-gp/ABCB1)-mediated drug efflux. The transport cycle proceeds from an inward-open state, in which the substrate binds the transmembrane domains (TMD_1_/TMD_2_), through an occluded state coupled to ATP binding and dimerisation of the nucleotide-binding domains (NBD_1_/NBD_2_), to an outward-open state that releases substrate into the extracellular space. P-gp expression and efflux activity are up-regulated by the tumour microenvironment (hypoxia, acidosis, inflammatory cytokines) and by oxidative stress, acting through redox- and stress-responsive transcription factors (HIF-1α, NF-κB, Nrf2) that increase ABCB1 transcription [73,86,88,97].

The transition states of P-gp kinetic mechanism are basal ATP-ase activity and coupled drug transport. In these two states, conformational reorientation of P-gp occurs. Basal ATP-ase activity is a mechanical property of P-gp and constitutes the basal cycle, a separate pathway from ATP hydrolysis (Figure 2). In the basal cycle, no substrate can pass through P-gp. The NBD1 and NBD2 sites for drugs are in the “off sites” conformation on the outer surface of the membrane. Proteolytic cleavage of the linker region, which binds the NH2 and COOH regions of the P-gp protein, generates an active form of P-gp, with drug-independent or drug-dependent ATPase activity. P-gp adopts two conformations: one mediates the basal reaction and the other the drug-stimulated reaction, with the two conformations representing two distinct states [95,96,97].

Initially, the drug will bind to the high-affinity ‘on sites’, which are found on the inner surface of the membrane. After passing through the high-energy transition state, the drug will be released on the other side of the membrane. Most P-gp substrates have high hydrophobicity and positive electrical charge. For this reason, P-gp-interacting drugs will bind in the phospholipid bilayer and concentrate on the inner face of the cell membrane. Drug expulsion suggests a “hydrophobic vacuum cleaner” mechanism. Transmembrane movement of the drug via P-gp is a flip-flop mechanism. Binding of the drug to the membrane phospholipid layer is a major factor in the recognition of this substrate by P-gp. Some studies show that cholesterol levels influence P-gp localisation. On the same principle, studies show that increased intracellular and transmembrane cholesterol levels can increase P-gp activity [73,98].

In addition to its well-characterized efflux activity, P-gp regulation appears to be interconnected with broader cellular signaling pathways and metabolic states. Recent literature emphasizes that transporter expression can be influenced by inflammatory mediators, oxidative stress, and metabolic regulators. From a translational perspective, understanding these biological pathways is essential, particularly in the context of cancer theranostics, where molecular targeting strategies are increasingly explored [55,99,100,101].

### 2.4. P-gp Substrates in Cancer

Substrate recognition by P-gp primarily occurs within the transmembrane domains (TMDs), while ATP binding and hydrolysis take place at the two nucleotide-binding domains (NBDs). Structural studies indicate that substrate binding within the TMD induces conformational changes that are transmitted to the NBDs, promoting ATP hydrolysis at the two catalytic sites located approximately 40 Å apart. Recent cryo-electron microscopy (cryo-EM) studies show that substrate binding causes a shortening of the distance between NBD1 and NBD2 [102,103]. Mechanistically, substrate binding does not contract this distance directly; rather, it acts through allosteric coupling. Engagement of the substrate by the aromatic and hydrophobic residues lining the TMD cavity is relayed, via the transmembrane helices and the intracellular coupling helices that dock into grooves on the NBD surface, into a reorganization of the NBDs that increases their affinity for Mg-ATP and lowers the energetic barrier to their association. Binding of two Mg-ATP molecules then drives the NBDs to associate into a head-to-tail nucleotide-sandwich dimer, in which each of the two composite catalytic sites is formed by the Walker A and Walker B motifs of one NBD together with the LSGGQ signature motif of the opposing NBD; closure of this dimer is what shortens the NBD1-NBD2 distance observed by cryo-EM. This conformational change is, in turn, mechanically relayed back through the coupling helices to switch the transmembrane domains from the inward-facing to the outward-facing state, thereby lowering substrate affinity and releasing the drug toward the extracellular side, after which ATP hydrolysis and the release of ADP and inorganic phosphate disengage the NBDs and reset the transporter to its inward-facing, high-substrate-affinity conformation. Accordingly, the cryo-EM observation of NBD approximation upon substrate binding reflects substrate-induced priming of the ATP-driven dimerization step rather than a direct, substrate-mediated contraction of the two domains. It is evident that P-gp possesses a high and versatile capacity to bind different substances on numerous and sometimes overlapping binding sites, which facilitates interaction with lower or higher molecular weight molecules, or even several at the same time. P-gp substrates are often fat-soluble or amphiphilic in nature. The media-binding region of P-gp is based on either aromatic or hydrophobic residues, which create van der Waals bonds with the molecules they come into contact with. At the same time, we can also observe a limited number of polar residues (e.g., Gln343, Gln721), in addition to tyrosine and tryptophan aromatic residues, oriented towards the inner part of the P-gp. These polar residues can mediate hydrogen bonding interactions with various ligands [104].

A major goal of P-gp structural studies has been the understanding how a single transporter can recognize such a broad spectrum of chemicals. In recent studies using photoaffinity or derivative and mutagenic scanning procedures with thiol-active reagents, it has been shown that the surface where the substrate will be found upon binding corresponds to a specific site between the two TMD chains. Early pharmacological studies suggested the presence of multiple functional drug-binding regions within the P-gp cavity, commonly referred to as the H-site (Hoechst site), R-site (Rhodamine site) and P-site (Prazosin site). However, more recent structural investigations indicate that these sites likely represent overlapping regions within a large polyspecific drug-binding pocket.

However, these functional characterizations failed to provide accurate or relative binding information to the specific position where the substrate bound. Therefore, the successful co-crystallization of P-gp with various cyclic peptide ligands has provided unprecedented details of polyspecific binding sites [105].

#### Development of P-gp Inhibitors and Related Challenges

In recent years, there has been a great deal of interest in the development of new molecules with P-gp inhibitory potential. However, many of these molecules have not had the expected success [106,107,108].

An obvious challenge comes from the potential toxicity that P-gp inhibitors could exert, particularly due to the spread of P-gp in many tissues in the human body and its cellular protective role. In order to minimize the toxic potential on healthy tissues, methods of targeted inhibitor action on P-gp overexpressed on the surface of tumor cells were developed. Another major challenge of existing P-gp inhibitors is their low potency and low specificity. First-generation P-gp inhibitors were not developed specifically to decrease MDR in cancer, so they exert other undesirable pharmacodynamic actions. For example, verapamil, one of the first P-gp inhibitors developed in clinical trials, is a calcium channel blocker and its affinity for P-gp is only 10μM. Cyclosporin A, another P-gp inhibitor with low affinity, has a strong immunosuppressive effect. The low affinity of these molecules means that high doses are needed to achieve the desired effect and this can lead to a high degree of adverse reactions. Moreover, concomitant administration of these MDR inhibitors increased the plasma concentration of anticancer drugs by interfering in their metabolism and excretion pathways [109,110].

Newer MDR inhibitors, such as Tariquidar or Zosuquidar, have been optimised for increased potency (EC50 < 100 nM) and specificity. Several of these molecules show very low pharmacokinetic interactions with anticancer drugs compared to previous ones. However, there are conflicting data in the literature on the mechanism of action of Tariquidar. Some data claim that it is an ATP-ase stimulator, while other studies support its inhibitory action on ATP-ase. Moreover, it is not yet elucidated whether or not this drug also acts as a substrate for P-gp. In conclusion, the potency and specificity of currently known P-gp inhibitors are low compared to many drugs in clinical use at this time [111,112].

Another challenge comes from the P-gp structure. Current studies give less accurate information on where the drug will bind compared to studies for receptors or enzymes, making the development of P-gp inhibitors even more challenging [113,114,115]. A few bivalent inhibitors have been developed in different studies to interact with multiple P-gp binding sites, showing higher potency compared to monovalent inhibitors. Another question, for which there is still no answer, is the specific P-gp conformation that a potential inhibitor drug should target. This area of study is still incompletely explored and, for the development of agents with maximal affinity for P-gp, more complete models describing the binding sites of this efflux protein would be needed [116].

Currently, MDR remains a major obstacle in cancer treatment. Therefore, discovering new P-gp inhibition strategies that take these obstacles into account and try to minimize them would be a very important step in developing more effective cancer therapies (Table 1).

## 3. Biochemical Mechanisms of P-gp Inhibition

### 3.1. Selective COX-2 Inhibitors

N-[2-(cyclohexyloxy)4-nitrophenyl]-methanesulfonamide (NS-398) is a specific inhibitor of COX 2 and P-gp. Studies show that COX-2 is up-regulated in a large number of cancers, facilitating angiogenesis, metastatic capacity and anti-apoptotic activity. Other selective COX-2 inhibitors, such as meloxicam or rofecoxib, have shown involvement in decrease multi-drug resistance by reducing the expression of ABC transport proteins, including P-gp. Also, the treatment with NS-398 monotherapy showed no obvious effect on reducing tumor cell proliferation in breast cancer, but it is able to prevent or even reduce the onset of chemoresistance in breast cancer [127]. In a study in which breast tumor cells were given low doses of Dox (doxorubicin) to develop resistance, increased levels of both P-gp and COX-2 were noted. On these cells, the treatment with NS-398 showed down-regulation of P-gp and COX-2, with an implicit decrease in the resistance phenomenon. This phenomenon occurred for cells treated with Dox for 10 days. For those treated with Dox for 2 months, the resistance phenomenon was irreversible, with NS-398 no longer able to exert its COX-2 and P-gp inhibitory effects. Therefore, it is believed that Dox and NS-398 combination therapy, from the outset of treatment, may bring a beneficial effect in delaying or even completely preventing COX-2 and P-gp-mediated Dox resistance. Thus, through P-gp inhibition, a significantly higher amount of chemotherapeutic agent was accumulated and exerted its effect for a longer duration in the breast cancer tumor cell in vitro [128,129].

Paradoxically, the phytochemical kingdom that has historically served as the primary source of clinically employed antineoplastic agents: vincristine and vinblastine isolated from *Vinca rosea*, the taxane paclitaxel derived from *Taxus brevifolia*, and the camptothecin analogs originating from *Camptotheca acuminata* simultaneously harbor a chemically diverse repertoire of secondary metabolites endowed with MDR-modulatory and P-gp inhibitory properties [130,131]. Phytocomplexes comprising alkaloids, flavonoids, terpenoids, and polyphenolic constituents have been demonstrated to exert pleiotropic interference with tumorigenic mechanisms: some, such as berberine (an isoquinoline alkaloid), induce apoptosis via mitochondrial membrane depolarization and caspase-3/9 activation while concurrently downregulating ABCB1 expression at the transcriptional level; others, including curcumin and resveratrol, suppress oncogenic transcription factors implicated in both carcinogenesis and the induction of MDR. Furthermore, synergistic phytochemical combinations, either as standardized botanical extracts or as semi-synthetic phytocomplex formulations, have been reported to sensitize MDR tumor cell lines to conventional chemotherapeutics through allosteric or competitive inhibition of P-gp ATPase activity, modulation of membrane fluidity, and suppression of the signaling networks governing ABC transporter gene transcription, thereby positioning plant-derived molecules as compelling scaffolds for the rational design of adjuvant MDR-reversal strategies [132,133,134,135,136]. Increasing evidence suggests that oxidative stress and antioxidant defense systems play a critical role in cancer progression and in the modulation of MDR mechanisms. Natural bioactive compounds derived from plant sources have attracted significant scientific interest due to their antioxidant, anti-inflammatory and potential chemomodulatory properties. In particular, phytocomplexes rich in polyphenolic compounds extracted from spontaneous flora have demonstrated significant antioxidant activity and the capacity to influence cellular redox balance [137,138,139,140,141,142,143,144]. Such biological properties may contribute to the modulation of oxidative stress-dependent signaling pathways involved in tumor progression and drug resistance.

### 3.2. Quercetin

Quercetin is a ubiquitous flavonoid found in many families of medicinal plants. This substance is known for its potential to inhibit the development of cancer cells of various types, such as liver, lung, breast, and colorectal and gastric cancer. Animal studies have shown that this flavonoid acts selectively on cancer cells without causing damage to surrounding healthy cells. It may also increase the sensitivity of cancer cells to chemotherapy, a dose-dependent action, which may explain Quercetin’s role in combating drug resistance. The main mechanisms are inhibition of Y-box binding protein 1 (YB-1) and increased chemosensitivity via P-gp inhibition in breast cancer. By inhibiting YB-1, Quercetin can be a successful agent in eliminating cancer stem cells, whose growth is YB-1-mediated by nuclear translocation [129,145]. In a study that focused on analyzing the impact of Quercetin on MDR in breast cancer, two cell media, denoted as MCF-7 and MCF-7/dox, were used. The MCF-7 medium used breast cancer cells, while the MCF-7/dox medium was pre-treated with a low amount of Dox, which influenced the up-regulation of P-gp and the establishment of the resistance phenomenon. The two media were treated with the chemotherapeutic agents Dox, Paclitaxel and Vincristine, alone or in combination with Quercetin. The results showed that the flavonoid, used alone, had a limited effect on cell proliferation. However, notable results appeared when combined with the 3 previously mentioned drugs, where quercetin increased the antitumor activity of Dox by 1.38× in the MCF-7 cell line and by 1.73× in the MCF-7/dox cell line, quercetin increased the antitumor activity of Paclitaxel by 1.68× in the MCF-7 cell line and by 2.45× in the MCF-7/dox cell line, quercetin increased the antitumor activity of Vincristine by 1.21× in the MCF-7 cell line and by 3.15× in the MCF-7/dox cell line. These results demonstrated the inhibitory action of Quercetin on YB-1 and P-gp protein, responsible for the development of drug resistance to conventional chemotherapy in breast cancer [129,145,146,147].

Although curcumin, quercetin and resveratrol modulate P-gp expression and activity in experimental systems, their clinical translation is constrained by well-documented pharmacokinetic limitations. Curcumin displays very low oral bioavailability (frequently <1%), owing to poor aqueous solubility, extensive first-pass glucuronidation and sulfation, and rapid systemic elimination; resveratrol and quercetin are likewise subject to rapid conjugation and low plasma exposure. Consequently, the intracellular concentrations required to inhibit P-gp in vitro are difficult to attain at tolerated oral doses in vivo. Pronounced inter-individual variability—arising from diet, microbiota-dependent metabolism and polymorphisms in conjugating enzymes—further complicates dosing, and robust randomised clinical evidence for a chemosensitising effect is currently lacking. These compounds are therefore best regarded as mechanistically interesting but pharmacokinetically challenged candidates, whose clinical utility will depend on advanced formulation strategies (e.g., nanoencapsulation) and adequately powered trials, rather than as established modulators.

### 3.3. Pluronic P85

Pluronic acid-based compounds are examples of polymers with remarkable capabilities and major adjuvant potential in the reduction of MDR, with their high biocompatibility and flexibility of the polymer chain being of great importance. These pluronic compounds are amphiphilic A-B-A copolymers, containing polyethylene-oxide hydroxyl and polypropylene-oxide hydrophobic components. In resistant cancer, the use of Pluronic P85 has been shown to restore the sensitivity of cancer cells to antineoplastic treatment. Due to the amphiphilic lipid-like structure, these compounds can diffuse into cell membranes and successfully inhibit efflux transporters such as P-gp, leading to increased intracellular accumulation of drugs [148]. In addition, pluronic compounds have been shown to increase intracellular ATP depletion, which significantly decreases P-gp activity. In a study of breast carcinoma cell lines, previously treated with Dox, Pluronic P85 demonstrated preventive capabilities for the development of resistance to chemotherapy. Initially, cells were subjected to increasing concentrations of Dox with or without Pluronic P85 for 305 days in vitro. In the cell line treated with Dox, tumor cell growth was observed compared to the cell line treated with the Dox-P85 combination, where tumor cells did not acquire resistance and were annihilated, mainly based on the cytotoxic action of Dox combined with P-gp and ABCB1 inhibition of the pluronic compound at 10 ng/mL concentration [148,149]. Other studies using DNA microarray analysis showed that P85 led to the abolition of alterations in genes involved in drug metabolism, apoptosis, stress response, transcriptional factors and tumorigenesis. In conclusion, P85 co-administration led to increased therapeutic efficacy in breast cancer patients. Similar effects have been observed in leukaemia, in vitro and in vivo, based on the same mechanism of P-gp inhibition [150,151].

### 3.4. The GO-MBs-Dox Nanosystem

The GO-MBs-Dox nanosystem is based on a nano-encapsulation structure of Dox in a graphene oxide (GO) matrix modified using 2 molecules of matrix DNA (MBs). Upon interaction of this nanosystem with resistant breast cancer tumor cells, Dox is released into the endocytosomes and MBs is hybridized with the DNA sequences of the target cell. In this way, these MBs exert their P-gp inhibition effect and thereby reduce the efflux of Dox out of the cell, leading to its accumulation in the cancer cell and exerting its cytotoxic effect. Compared to other therapeutic strategies for P-gp inhibition, the strategy of gene silencing combined with chemotherapy is the most economical and effective. Confocal fluorescence and flow cytometry data showed that Dox accumulation in breast cancer cells was drastically increased in the GO-MBs-Dox therapeutic system. In addition, PCR assays showed that MDR1 mRNA resistance gene expression in the presence of GO-MBs-Dox agent was much lower than usual [152,153,154,155,156].

### 3.5. Ixabepilone

Ixabepilone was the first epothilone derivative approved for clinical use by the FDA in October 2007. Current clinical data highlight the role of epothilone derivatives in treating taxane-resistant forms of cancer, with Ixabepilone shown to be unaffected by established chemoresistance mechanisms [157,158,159]. This drug is used as monotherapy in patients with advanced metastatic breast cancer in whom anthracyclines, taxanes or capecitabine have failed or in combination with capecitabine in patients for whom atracicline or taxane therapy has failed due to the acquisition of resistance. Ixabepilone was developed specifically for patient categories with chemotherapy-resistant tumors, as it has reduced susceptibility to other MDR mechanisms.

The antineoplastic properties of this drug derive from its ability to bind to and block tubulin fragments in the microtubules of cancer cells, resulting in the blockade of mitosis in the G2/M phase and ultimately apoptosis. The efficacy of taxanes is known in the chemotherapeutic treatment of breast cancer, but the main problem comes from the rapid onset of MDR to these drugs. This has led to the development of new drugs with taxane-like mechanisms, but with less potential for resistance. This is how the epothilone derivatives emerged. Although they bind beta-tubulin, similar to taxanes, crystallographic studies have shown that the binding of epothilone derivatives is geometrically different from that of taxanes, which would explain their lower susceptibility to resistance development. Mutations at the beta-III-tubulin level similarly fail to induce resistance to Ixabepilone. Moreover, P-gp up-regulation in breast cancer cells showed a much lower influence on the epothilone derivatives compared to the taxanes. Thus, these derivatives offer a much more effective therapeutic alternative to conventional chemotherapy in terms of MDR [158,159,160].

### 3.6. NAB-Paclitaxel

NAB stands for “nanoparticle albumin-bound” and is an innovative strategy to deliver paclitaxel in a form that avoids hypersensitivity reactions and toxicity associated with carrier solvents, with more selective and effective activity on breast cancer tumor cells. It consists of paclitaxel nanoparticles reversibly bound to human serum albumin, giving it higher transport and distribution capabilities. In fact, given that tumor cells in advanced stages produce secreted protein acidic and rich in cysteines to bind and feed on blood albumin, NAB-Paclitaxel, having human albumin in its composition, is recognized by the tumor cell and selectively binds to it, practically inducing high selectivity to this drug and accumulation of high concentrations at the site of action [161]. In addition to this, the NAB platform eliminates the disadvantage of using toxic solvents due to hydrophobic binding of albumin, which offers the chance of infusing higher concentrations of paclitaxel compared to standard therapy without prior medication [162].

NAB-Paclitaxel was developed as a more effective and tolerable therapeutic alternative to conventional therapy. In phase 2 and 3 trials, the efficacy and safety profile of NAB-Paclitaxel for the treatment of metastatic breast cancer were examined, even in women previously treated with anthracyclines. These studies have shown much better overall response rates and progression free survival data than conventional Paclitaxel therapy [163,164,165].

### 3.7. Bexarotene

Bexarotene is also known as LGD1069 or Targretin. It is a selective ligand on retinoid X receptors, used for its chemopreventive and chemotherapeutic action in mouse models of breast cancer. Its main adverse effects are hypertriglyceridemia and hypothyroidism, which can be controlled using other classes of drugs [166]. Bexarotene has shown a high clinical potential for combination with other cytotoxic drugs due to its non-overlapping safety profile, providing a more manageable safety benefit and adverse reaction profile compared to other combinations. Combination with Bexarotene can prevent or limit MDR, in vivo or in vitro, in different types of cancer [167].

Bexarotene exerts its modulatory effects on multidrug resistance through several complementary mechanisms that have been collectively validated in mouse models. At the molecular level, resistant cells characteristically overexpress ABCB1 and P-gp on their surface; however, following treatment with Bexarotene in combination with conventional chemotherapeutic agents, the expression of these efflux transporters becomes nearly undetectable, thereby attenuating one of the most critical determinants of the MDR phenotype. Beyond its capacity to downregulate transporter expression, the combination of Bexarotene with cytotoxic agents has further demonstrated the ability to reduce both the metastatic potential and the angiogenic activity of tumor cells, suggesting a broader interference with the pro-tumorigenic microenvironment that sustains resistant clones. Additionally, Bexarotene has been shown to preserve genomic integrity within tumor cells by limiting the accumulation of spontaneous mutations, a mechanism that may attenuate the acquisition of new resistance traits over the course of treatment. Collectively, these findings position Bexarotene as a pleiotropic modulator of drug resistance, and its clinical relevance extends beyond this context, as the compound is also employed in combinatorial regimens for other malignancies, including metastatic non-small cell lung cancer [166,168].

The pharmacological strategies developed to overcome P-gp-mediated multidrug resistance have evolved considerably over the past decades, spanning from early direct inhibitors to sophisticated nanomedicine platforms. As summarized in Table 2, these approaches can be broadly categorized into seven strategic classes, each characterized by a distinct mechanism of action and a specific set of clinical or translational challenges. First-generation inhibitors, such as verapamil and cyclosporin A, pioneered the concept of direct P-gp transport blockade but were quickly recognized to suffer from poor selectivity and unfavorable pharmacokinetic interactions with co-administered cytotoxic agents. Second-generation inhibitors, exemplified by valspodar (PSC-833), addressed potency limitations, yet introduced new concerns regarding toxicity and drug–drug interactions that hampered their clinical advancement. The third generation, including tariquidar and zosuquidar, achieved high selectivity for P-gp but ultimately demonstrated limited efficacy in randomized clinical trials, highlighting the complexity of MDR beyond transporter inhibition alone. In parallel, interest in natural compounds such as quercetin and curcumin has grown due to their capacity to downregulate P-gp expression and modulate upstream signaling pathways, although inconsistent bioavailability remains a critical obstacle to their therapeutic exploitation. At the nanotechnology frontier, polymer-based delivery systems, most notably Pluronic P85, have been shown to deplete intracellular ATP and inhibit transporter function, while nanomedicine platforms incorporating graphene oxide nanosystems and lipid nanoparticles offer the dual advantage of enhanced intracellular drug delivery and gene-silencing capabilities; however, both approaches still require extensive clinical validation before translation into routine oncological practice. Finally, the development of novel anticancer agents inherently less susceptible to P-gp efflux, such as ixabepilone and nab-paclitaxel, represents an orthogonal strategy that circumvents the transporter rather than inhibiting it, though cost constraints and narrow approved indications currently limit their widespread use. Taken together, this landscape underscores the need for combinatorial or patient-stratified approaches that integrate mechanistic insights with clinically actionable solutions.

The repeated clinical failure of P-gp inhibitors is itself instructive. First-generation agents (verapamil, cyclosporin A) lacked selectivity and produced dose-limiting cardi-ovascular and immunosuppressive toxicity. The second-generation agent valspodar (PSC-833) inhibited CYP3A4, thereby altering the pharmacokinetics of co-administered cytotoxics and necessitating chemotherapy dose reductions that both confounded efficacy assessment and increased toxicity. Third-generation inhibitors, although highly selective and largely devoid of CYP3A4 interaction, also failed to improve outcomes: in the ran-domised, placebo-controlled ECOG 3999 phase III trial, zosuquidar did not improve survival in older patients with acute myeloid leukaemia, and its development was subsequently discontinued, while phase III evaluation of tariquidar with paclitax-el/carboplatin in non-small-cell lung cancer was likewise unsuccessful. Several converging factors explain these disappointments: the futility of blocking a single transporter when redundant pumps (ABCG2, the ABCC/MRP family) and non-transporter mechanisms provide compensatory resistance; the near-absence of predictive biomarkers and of patient selection based on tumour P-gp status; pronounced intratumoral and interpatient heterogeneity; and the narrow therapeutic window created by inhibition of physiological P-gp at the blood–brain barrier and excretory organs. These lessons argue for biomarker-guided patient stratification and combinatorial strategies rather than transporter inhibition alone. The principal clinical trials of P-gp inhibitors across the three generations are summarised in Table 3, whereas the representative nanomedicine and polymeric strategies and the clinical-evidence status of the main phytochemicals discussed are presented in Table 4 and Table 5, respectively.

**Table 3:** Major clinical trials of P-gp inhibitors, by generation.

Agent	Generation	Setting	Trial Phase	Principal Outcome/Reason for Limitation
Verapamil	First	Haematologic and solid tumours	Early clinical	Calcium-channel blocker; low P-gp affinity (~10 µM); cardiovascular toxicity at MDR-reversal doses
Cyclosporin A	First	Haematologic malignancies	Early clinical	Potent immunosuppression and pharmacokinetic interactions; poor selectivity
Valspodar (PSC-833)	Second	AML and others	Phase II/III	CYP3A4 inhibition altered cytotoxic pharmacokinetics, forcing dose reductions; no clear benefit
Zosuquidar	Third	Older AML/high-risk MDS	Phase III (ECOG 3999)	Did not improve survival; development discontinued
Tariquidar	Third	NSCLC (+paclitaxel/carboplatin)	Phase III (NCT00042302)	Unsuccessful; no efficacy gain despite high selectivity
Elacridar	Third	Solid tumours	Early phase/research	Mainly a research and imaging tool; limited clinical development

**Table 4:** Representative nanomedicine and polymeric strategies against P-gp-mediated efflux.

Platform	Cargo/Function	Mechanism Relative to P-gp	Highest Stage of Development
Pluronic P85 (block copolymer)	Chemosensitiser	ATP depletion and membrane fluidisation, reducing transporter activity	Preclinical/early clinical
Lipid nanoparticles	Cytotoxic drug ± siRNA	Endocytic uptake bypasses efflux; ABCB1 gene silencing	Preclinical; clinical for related platforms
Graphene-oxide nanosystems	Drug + gene	Enhanced intracellular delivery and co-delivery/silencing	Preclinical
Polymeric micelles/drug conjugates	Cytotoxic drug	Altered intracellular trafficking and sustained release	Preclinical/early clinical

**Table 5:** Clinical-evidence status of the principal phytochemicals discussed.

Compound	Proposed Mechanism on P-gp	Highest Level of Evidence	Key Limitation
Curcumin	Down-regulates ABCB1 expression and inhibits efflux activity	In vitro and animal; small clinical studies	Very low oral bioavailability (<1%); extensive first-pass metabolism
Quercetin	Modulates P-gp activity and expression	In vitro and animal	Variable absorption; rapid conjugation; limited clinical data
Resveratrol	Modulates P-gp activity and upstream signalling	In vitro and animal; few clinical studies	Low bioavailability; rapid metabolism; inconclusive results

## 4. Discussion

Despite these encouraging advances, several challenges remain in translating P-gp-targeted strategies into clinical practice. One major limitation arises from the physiological role of P-gp in protecting vital organs such as the intestine, liver, kidney, and blood–brain barrier from xenobiotic accumulation. Systemic inhibition of this transporter may therefore lead to undesirable pharmacokinetic interactions and increased toxicity. Consequently, future therapeutic approaches should aim to achieve selective modulation of P-gp activity within tumor tissues, rather than global inhibition throughout the body.

From a broader scientific perspective, the study of multidrug resistance highlights the importance of integrating molecular pharmacology, systems biology, and advanced drug delivery technologies. MDR is increasingly recognized as a multifactorial process involving interactions between transporter expression, metabolic adaptation, tumor microenvironment, and genetic heterogeneity. Advances in computational modeling, molecular dynamics simulations, and machine-learning approaches for predicting transporter–drug interactions may significantly accelerate the development of more effective modulators of P-gp activity.

Future research should therefore focus on several key directions. First, high-resolution structural and computational studies are required to better characterize the conformational states of P-gp and identify novel binding pockets suitable for selective inhibition. Nevertheless, nanomedicine and targeted delivery platforms may enable selective transporter modulation specifically within tumor cells. Also, deeper investigation of the interplay between oxidative stress pathways, metabolic regulation, and ABC transporter expression may reveal new therapeutic targets capable of overcoming MDR.

Overall, a comprehensive understanding of P-gp biology and its role in multidrug resistance remains essential for the development of next-generation anticancer therapies. By integrating transporter modulation strategies with advances in targeted drug delivery and precision oncology, it may become possible to significantly improve therapeutic efficacy and reduce treatment failure in patients with drug-resistant cancers.

### 4.1. MDR Plasticity Beyond P-gp: Other Resistance Mechanisms

Although P-gp is the prototypical efflux pump, clinical MDR is multifactorial, and its plasticity extends well beyond a single transporter. Other ABC transporters contribute substantially, notably ABCG2 (BCRP) and the ABCC/MRP family (particularly MRP1/ABCC1 and MRP2/ABCC2), which efflux overlapping and distinct substrate sets and can compensate when P-gp is inhibited. Beyond efflux, resistance is sustained by evasion of apoptosis (for example, up-regulation of Bcl-2-family proteins and p53 dysfunction), by enhanced DNA-damage repair underlying platinum resistance, and by cancer stem cells, which combine relative quiescence with high ABCG2 expression (the ‘side-population’ phenotype) and intrinsic drug tolerance. Underpinning these programmes, metabolic reprogramming maintains the ATP supply and redox buffering on which both active efflux and cell survival depend. This broader view substantiates the concept of MDR plasticity emphasised in the title and clarifies why single-target strategies have limited durability.

### 4.2. Limitations and Open Challenges

Several limitations of the current MDR research landscape temper the optimism of this review. There are, as yet, no validated predictive biomarkers that reliably identify patients whose resistance is genuinely P-gp-driven and who might therefore benefit from transporter modulation. Intratumoral and interpatient heterogeneity means that resistance mechanisms differ between lesions and evolve under treatment pressure. Pre-clinical findings have repeatedly failed to translate into clinical benefit, reflecting both biological redundancy and the methodological limitations of model systems. Combination regimens and nanomedicine products face substantial regulatory hurdles, and the cost and narrowly approved indications of newer agents such as ixabepilone and nab-paclitaxel constrain their real-world adoption. These constraints should be borne in mind when interpreting the strategies discussed herein.

## 5. Conclusions and Future Perspectives

**Figure 3:**
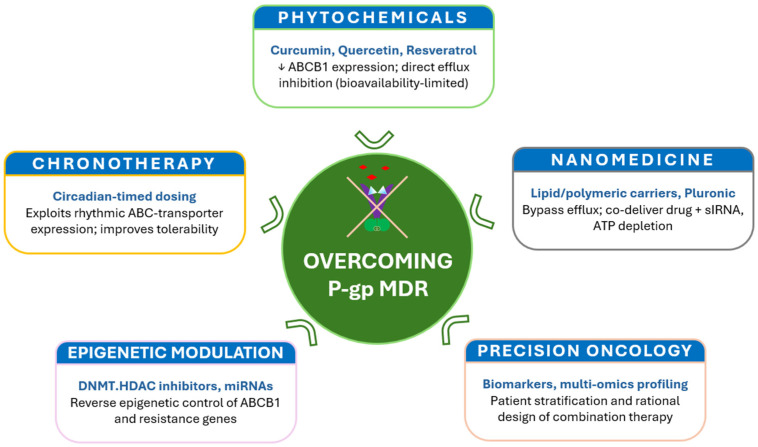
Integrative overview of multimodal strategies to overcome P-gp (ABCB1)-mediated multidrug resistance. Five complementary approaches (bioavailability-limited phytochemicals, nanomedicine-based delivery, circadian-timed chronotherapy, epigenetic modulation, and biomarker-guided precision oncology) converge on the common goal of restoring chemosensitivity. The schematic emphasizes that durable reversal of MDR is more likely to arise from rational combination of these modalities than from any single strategy [11,134,145,146,148,171,172,173,174,175,176,177,178,179,180,181,182,183].

The evidence reviewed here indicates that MDR, driven substantially—though not exclusively—by the overexpression of P-gp (ABCB1) and related ATP-binding cassette efflux transporters, remains among the most challenging and clinically consequential obstacles to the curative treatment of malignant disease. Decades of mechanistic investigation have progressively elucidated the molecular architecture of MDR, revealing a biological system of remarkable plasticity, redundancy, and adaptive capacity. It is precisely this adaptive complexity that has rendered single-target pharmacological approaches insufficient and has catalyzed the emergence of integrative, multimodal therapeutic frameworks that are now redefining the landscape of oncological research and clinical strategy (Figure 3). The clinical failure of first-, second-, and third-generation P-gp inhibitors, from verapamil and cyclosporin A to tariquidar and elacridar, despite their compelling preclinical efficacy, has imposed a lesson upon the field: the pharmacokinetic and pharmacodynamic entanglement of potent ABC transporter modulators with their cytotoxic substrates creates a therapeutic space of unacceptable toxicity and unpredictable drug–drug interactions. Yet, rather than signaling a conceptual dead end, these failures have functioned as productive constraints, directing scientific attention toward more nuanced biological targets and more sophisticated delivery strategies. The transition from reversing MDR through brute-force transporter inhibition toward exploiting the molecular vulnerabilities that co-arise with the MDR phenotype, so-called collateral sensitivity, exemplifies this paradigm shift [169,170].

Among the most scientifically productive and translationally compelling domains to have emerged from MDR research is the systematic investigation of plant-derived secondary metabolites as modulatory agents. The phytochemical kingdom, which has historically furnished the majority of clinically deployed antineoplastic scaffolds, simultaneously harbors a chemically diverse repertoire of compounds capable of interfering with MDR at multiple mechanistic nodes. Alkaloids such as berberine, piperine, and the bis-indole alkaloids of *Catharanthus roseus*, polyphenolic constituents including curcumin, resveratrol, quercetin, and epigallocatechin-3-gallate, and terpenoid complexes such as those isolated from *Artemisia annua* and *Taxus* species have been demonstrated, across a substantial body of evidence, to modulate P-gp ATPase activity, suppress the transcriptional regulators of ABCB1 expression, and sensitize MDR tumor cell lines to conventional chemotherapeutic agents [133,184,185,186,187].

From a nutritional standpoint, growing evidence supports the concept of dietary chemosensitization: the regular consumption of bioactive phytochemicals through food matrices, cruciferous vegetables (sulforaphane, indole-3-carbinol), citrus flavonoids (naringenin, hesperidin), and curcuminoids derived from *Curcuma longa*, may modulate the constitutive expression of efflux transporters in tumor-prone tissues, offering a preventive dimension to the MDR problem that extends beyond the therapeutic window of active disease [16,21,134,188,189,190]. The recognition that the gut microbiome critically shapes the bioavailability and metabolic transformation of dietary polyphenols into pharmacologically active aglycones has further broadened the nutritional axis of MDR modulation, introducing the concept of microbiome–pharmacogenome interactions as a determinant of individual chemosensitivity [191,192,193].

The advent of nanomedicine has introduced a fundamentally different logic for overcoming MDR: rather than attempting to inhibit P-gp directly, nanoparticulate drug delivery systems exploit alternative cellular uptake pathways, principally endocytosis and macropinocytosis, that are categorically independent of ABC transporter-mediated efflux [8,153,194]. Liposomes, solid lipid nanoparticles, polymeric nanoparticles, dendrimers, carbon nanotubes, and inorganic nanocarriers have each been engineered to encapsulate cytotoxic payloads and deliver them intracellularly via endosomal trafficking routes, thereby bypassing the plasma membrane-localized efflux machinery entirely. Crucially, pH-responsive and redox-responsive nanocarriers are capable of exploiting the specific physicochemical environment of the tumor microenvironment, characterized by acidosis, elevated reactive oxygen species, and overexpression of matrix metalloproteinases, to achieve controlled drug release precisely within the intracellular compartment where it is most therapeutically effective [121,195].

The co-encapsulation strategy, whereby a cytotoxic agent and a P-gp inhibitor or MDR-reversing phytochemical (e.g., curcumin, verapamil, tariquidar) are loaded into a single nanoparticulate carrier, represents a particularly elegant solution: the simultaneous intracellular delivery of both the substrate and the inhibitor at pharmacologically relevant molar ratios circumvents the systemic toxicity that has historically plagued co-administration approaches [196,197]. Stimuli-responsive nanosystems engineered for ligand-targeted delivery, employing transferrin, folate, hyaluronic acid, or tumor-specific antibody fragments as homing moieties, further refine the selectivity of this approach, reducing off-target accumulation in normal tissues that constitutively express ABC transporters in physiologically protective roles [198].

A dimension of MDR that has historically received insufficient attention, yet has accumulated a compelling body of mechanistic and clinical support, is the circadian regulation of ABC transporter expression and activity. Molecular chronobiology has established that the expression of ABCB1, ABCG2, and ABCC2 exhibits robust circadian oscillation in multiple organ systems, including intestinal epithelium, hepatocytes, and renal proximal tubule cells, driven by the transcriptional network constituted by CLOCK, BMAL1, PER, and CRY proteins [171,199,200]. In tumor tissues, the circadian clock is frequently disrupted, a phenomenon associated with accelerated disease progression and chemotherapeutic resistance, yet residual oscillatory capacity in transporter expression can be pharmacologically exploited through the strategic timing of drug administration. Chronotherapeutic protocols, in which cytotoxic agents are delivered at times of day corresponding to lowest point P-gp expression or peak DNA repair activity in normal tissues, have demonstrated measurable improvements in tolerability and, in several clinical trials, in tumor response rates [172]. The integration of individual circadian phenotyping, including wrist actigraphy, urinary melatonin profiling, and clock gene expression analysis in peripheral blood mononuclear cells, into patient stratification algorithms represents a frontier application of precision oncology that holds the promise of substantially augmenting the therapeutic index of existing chemotherapeutic regimens [173,174,175].

While chronomodulated delivery of agents such as 5-fluorouracil, oxaliplatin and irinotecan has been associated with improved tolerability and, in some studies, response in metastatic colorectal cancer, the evidence remains heterogeneous. Notably, the EORTC Chronotherapy Group trials yielded inconsistent, sex-dependent outcomes, with a survival benefit observed in men but not in women, in whom a possible detriment was reported. Optimal timing is not standardised, inter-individual differences in circadian phase are substantial, and routine clinical adoption remains limited. We therefore now characterise chronotherapy as a biologically compelling but not yet clinically validated strategy, whose advance will require biomarker-guided (circadian-phenotyped) and adequately powered randomised trials.

Beyond transporter inhibition and pharmacological evasion, contemporary MDR research has increasingly focused on the epigenetic determinants of ABCB1 overexpression. The methylation status of CpG islands within the MDR1 promoter, the acetylation state of histones at the ABCB1 locus, and the post-transcriptional regulation of P-gp expression by non-coding RNAs and several long non-coding RNAs collectively constitute an epigenetic regulatory layer that is both mechanistically tractable and pharmacologically accessible [68,176].

The concept of synthetic lethality, operationalized through the inhibition of DNA damage repair pathways selectively in MDR-expressing tumor cells, has opened an entirely orthogonal strategic axis: rather than reversing MDR, this approach exploits the metabolic and genomic vulnerabilities that co-segregate with the MDR phenotype, including elevated oxidative stress, altered mitochondrial membrane dynamics, and compromised autophagy regulation, in order to convert the MDR tumor cell’s adaptations into lethal liabilities [177,178].

A transformative development in cancer biology has been the recognition that the tumor microenvironment, comprising cancer-associated fibroblasts, tumor-associated macrophages, regulatory T cells, myeloid-derived suppressor cells, and the extracellular matrix, is not merely a passive bystander to MDR but an active contributor, secreting cytokines, growth factors, and exosomes that transcriptionally upregulate ABC transporter expression in tumor cells and confer paracrine chemoresistance upon adjacent cell populations [179,180]. The integration of MDR-modulating strategies with immune checkpoint blockade and with adoptive cell therapies such as CAR-T cells represents a convergence of two of the most dynamic fields in modern oncology, with the potential for synergistic effects that transcend what either modality can achieve in isolation [181]. P-gp has itself been identified on the surface of natural killer cells and cytotoxic T lymphocytes, where it plays a role in the cellular efflux of cytotoxic perforins and granzymes, a finding with direct implications for the interpretation of clinical immunotherapy outcomes in MDR-expressing tumors [182].

Before outlining these forward-looking directions, it is important to distinguish three tiers of maturity, so that aspiration is not mistaken for established practice. (i) Current clinical reality comprises approaches already in routine use or supported by controlled trials, notably the standard cytotoxic backbone and efflux-evading agents such as nab-paclitaxel and ixabepilone. (ii) Emerging approaches under active investigation, at early clinical or advanced preclinical stages, include co-encapsulating nanomedicine platforms, chemosensitising phytochemicals, epigenetic modulators, microbiome modulation, and chronotherapy. (iii) Long-term perspectives remain largely conceptual and are not yet supported by clinical evidence; these include comprehensive circadian phenotyping for routine patient stratification, real-time adaptive therapy guided by mathematical models of clonal evolution, and full multi-omics and artificial intelligence integration. The principles articulated below should be read with these distinctions in mind.

Looking forward, the overarching trajectory of MDR research converges on three defining principles that will shape the next decade of translational oncology: precision, integration, and adaptivity.

Precision demands the individualization of MDR-reversing strategies based on comprehensive tumor molecular profiling, encompassing whole-exome sequencing of ABC transporter gene families, epigenomic mapping of MDR1 regulatory regions, proteomic quantification of efflux pump density at the tumor cell surface, and single-cell transcriptomic characterization of clonal heterogeneity within the MDR tumor subpopulation. The deployment of liquid biopsy technologies for real-time, non-invasive monitoring of emerging MDR phenotypes during the course of treatment will transform the clinical management of resistance from a reactive to a pre-emptive discipline [183].

Integration requires the abandonment of reductionist, single-target paradigms in favor of rationally designed combination regimens that simultaneously address multiple determinants of MDR. Combining, for example, a nanoparticulate cytotoxic payload with a co-encapsulated epigenetic modifier and a chronotherapy-informed administration schedule calibrated to the patient’s individual circadian phenotype and microbiome composition could be a future approach. Such combinatorial complexity necessitates the application of computational network pharmacology, systems biology modelling, and artificial intelligence-driven drug combination optimization platforms to navigate a therapeutic space that is beyond the reach of conventional empirical approaches [116,185].

Adaptivity recognizes that cancer is an evolving dynamic system, and that any fixed therapeutic protocol will ultimately face evolutionary escape. The design of adaptive treatment strategies, in which the nature, dose, and timing of therapeutic interventions are dynamically adjusted in response to real-time biomarker feedback from the tumor, represents the logical extension of precision oncology into the temporal dimension. Mathematical models of clonal evolution under therapeutic selection pressure, integrated with clinical decision-support systems, may ultimately enable the scheduling of treatment protocols that maintain tumor control while actively suppressing the emergence of MDR clones through principles analogous to those employed in the management of infectious disease resistance [201,202].

The field of MDR research has arrived at a moment of exceptional scientific richness and translational opportunity. The molecular mechanisms of P-gp-mediated and non-P-gp-mediated resistance are understood with unprecedented resolution. A broad arsenal of modulating strategies: pharmacological, phytochemical, nutritional, nanotechnological, epigenetic, chronotherapeutic, and immunological has been characterized and, in many cases, validated in clinical settings; the technological infrastructure for precision oncology is now sufficiently mature to begin integrating these advances into clinically deployable frameworks. What remains is the sustained commitment of the scientific and clinical community to the synthesis of these converging streams of knowledge into therapeutic paradigms that are equal to the adaptive complexity of the adversary they seek to overcome.

## Figures and Tables

**Table 1:** Major P-gp substrates involved in multidrug resistance in cancer [117,118,119,120,121,122,123,124,125,126].

Drug Class	Representative Drugs	Mechanism of Action	Impact of P-gp Overexpression
Anthracyclines	Doxorubicin, Daunorubicin	DNA intercalation and inhibition of topoisomerase II	Reduced intracellular drug accumulation and decreased cytotoxic activity
Taxanes	Paclitaxel, Docetaxel	Stabilization of microtubules and inhibition of mitosis	Active efflux by P-gp reduces intracellular drug levels
Vinca alkaloids	Vincristine, Vinblastine	Inhibition of microtubule polymerization	Rapid drug export leading to decreased therapeutic response
Epipodophyllotoxins	Etoposide	Topoisomerase II inhibition	Efflux-mediated reduction of drug exposure
Tyrosine kinase inhibitors	Imatinib, Dasatinib	Inhibition of oncogenic signaling pathways	Reduced intracellular drug concentrations in resistant tumors
Other chemotherapeutics	Actinomycin D, Mitoxantrone	DNA damage and inhibition of transcription	P-gp mediated drug transport contributes to MDR

**Table 2:** Therapeutic strategies investigated for overcoming P-gp-mediated multidrug resistance [109,110,111,112,119,129,134,145,148,149,152,153,154,155,156,157,158,159,160,161,162,163,164,165].

Strategy	Examples	Mechanism	Current limitations	Level of Evidence	Development Stage
First-generation inhibitors	Verapamil, Cyclosporin A	Direct inhibition of P-gp transport activity	Low specificity and pharmacokinetic interactions	Early clinical trials	Discontinued
Second-generation inhibitors	Valspodar (PSC-833)	Increased potency against P-gp	Toxicity and drug–drug interactions	Phase II/III	Development halted
Third-generation inhibitors	Tariquidar, Zosuquidar	Highly selective P-gp inhibition	Limited clinical efficacy in trials	Phase III (negative)	Failed/discontinued
Natural compounds	Quercetin, Curcumin	Downregulation of P-gp expression and modulation of signaling pathways	Variable bioavailability	In vitro/animal	Preclinical
Polymer-based delivery systems	Pluronic P85	ATP depletion and inhibition of transporter activity	Limited clinical translation	In vitro/animal	Preclinical/early clinical
Nanomedicine approaches	Graphene-oxide nanosystems, lipid nanoparticles	Enhanced intracellular drug delivery and gene silencing	Need for further clinical validation	In vitro/animal	Preclinical (clinical for some LNPs)
Novel anticancer agents less susceptible to P-gp	Ixabepilone, Nab-Paclitaxel	Reduced efflux susceptibility	Cost and limited indications	Randomised clinical trials	FDA-approved

## Data Availability

No new data were created or analyzed in this study. Data sharing is not applicable to this article.
